# Evaluation of surrogate measures of insulin sensitivity - correlation with gold standard is not enough

**DOI:** 10.1186/s12874-018-0521-y

**Published:** 2018-06-26

**Authors:** Anna Rudvik, Marianne Månsson

**Affiliations:** 1 AstraZeneca, Pepparedsleden 1, Mölndal, 43153 Sweden; 2 Department of Urology, Institute of Clinical Sciences, Sahlgrenska Academy at the University of Gothenburg, Bruna Stråket 11B, Gothenburg, 41345 Sweden

**Keywords:** Correlation, Insulin sensitivity, Surrogate measure

## Abstract

**Background:**

Impaired insulin sensitivity is a key abnormality underlying the development of type 2 diabetes. Measuring insulin sensitivity is therefore of importance in identifying individuals at risk of developing diabetes and for the evaluation of diabetes-focused interventions. A number of measures have been proposed for this purpose. Among these the hyperinsulinemic euglycemic clamp (HEC) is considered the gold standard. However, as the HEC is a costly, time consuming and invasive method requiring trained staff, there is a need for simpler so called surrogate measures.

**Main message:**

A frequently used approach to evaluate surrogate measures is through correlation with the HEC. We discuss limitations with this method. We suggest other aspects to take into consideration, such as repeatability, reproducibility, systematic biases and discrimination ability. In addition, we focus on three frequently used surrogate measures. We argue that they are one-to-one transformations of each other, and therefore question the benefits of further comparison between them. They give the same results in all rank-based methods, for instance Spearman correlations, Mann-Whitney tests and receiver operating characteristic (ROC) analysis.

**Conclusions:**

We suggest investigating further aspects than correlation alone when evaluating a surrogate measure of insulin sensitivity. We recommend choosing one of the three surrogate measures HOMA-IR, QUICKI and FIRI for analysis of a clinical study.

**Electronic supplementary material:**

The online version of this article (10.1186/s12874-018-0521-y) contains supplementary material, which is available to authorized users.

## Background

The term insulin sensitivity refers to the body’s sensitivity to the effects of insulin and is an umbrella term for various physiological processes. Individuals with low insulin sensitivity require larger amounts of insulin in order to keep blood glucose stable. Impaired insulin sensitivity is a key abnormality underlying the development of type 2 diabetes as well as several other clinical states. Measuring insulin sensitivity is of importance in identifying individuals at risk of developing diabetes and to evaluate diabetes-focused interventions. A number of measures of varying complexity have emerged for this purpose. The hyperinsulinemic euglycemic clamp, HEC (described in de Fronzo et al. [1], among others) is considered the reference method for the measurement of insulin sensitivity and is referred to as the gold standard. In the clamp technique, insulin and glucose are both continuously infused into the bloodstream to maintain plasma insulin concentration at a constant high level and plasma glucose concentration at a constant basal level. *M*_*LBM*_, the mean glucose infusion rate reached at steady state normalized per kilogram lean body mass, is a measure of insulin sensitivity. The HEC is a costly, time consuming and invasive method requiring trained staff. There is consequently a need for alternative so called surrogate measures, especially for large scale epidemiological studies.

The surrogate measures are grouped into two families of indices, the OGTT-based indices (OGTT = oral glucose tolerance test) and the fasting indices (see Table 1). The OGTT-based indices (e.g. Matsuda, Stumwoll) are based on changes in plasma concentrations of insulin and glucose during an OGTT. Some of the fasting indices, e.g. the homeostasis model of insulin resistance (HOMA-IR), the quantitative insulin sensitivity check index (QUICKI) and the fasting insulin resistance index (FIRI), are based only on fasting plasma concentrations of glucose and insulin, while others include additional biomarkers. A more complete table of surrogate measures can be found in e.g. Otten et al. [2].

**Table 1:** Common surrogate measures with formulas

	Surrogate measure	Formula
OGTT-based indices	Stumwoll	18.3−0.271·*BMI*−0.0052·
		*I*_120_−0.27·*G*_90_
	Matsuda	$10,000\left (\sqrt {G_{0}\!\cdot \! I_{0}\!\cdot \! G_{mean}\cdot I_{mean}}\right)$
Fasting indices	HOMA-IR	(*G*_0_·*I*_0_)/22.5
	QUICKI	1/(log*G*_0_+log*I*_0_)
	FIRI	(*G*_0_·*I*_0_)/25
	Revised QUICKI	1/(log*G*_0_+log*I*_0_+log*NEFA*)
	Ratio of fasting insulin to glucose	*I*_0_/*G*_0_
	Ratio of fasting glucose to insulin	*G*_0_/*I*_0_

BMI, body mass index; *G*_0_, fasting gluc, glucose 90 min after administration of glucose; *G*_*mean*_, mean glucose during OGTT; *I*_0_, fasting insulin; *I*_120_, insulin 120 min after administration of insulin; *I*_*mean*_, mean insulin during OGTT; NEFA, non-esterified fatty acids

There is a wide range of publications discussing the pros and cons of the surrogate measures from various perspectives. Singh and Saxena [3], Gutch et al. [4], Otten et al. [2], among others, present overviews and descriptions of many of the surrogate measures. Borai et al. [5] give an excellent discussion on the choice of suitable measure from the perspective of the nature of the study. Depending on the purpose and situation, some publications concentrate on certain populations, while others attempt more general comparisons. The conclusion is often that one measure is the best choice for a specific population under study.

The most common way of evaluating surrogate measures appears to be by using correlation coefficients with the HEC ([6–14], among others). Other comparative analyses, such as ROC (receiver operating characteristic) analysis are also used (Rössner et al. [15] and Ruige et al. [16]). These methods are however less frequently used. In particular, the fasting surrogate indices HOMA-IR and QUICKI are often compared. Different authors propose that one or the other is the one to use, or even that both should be used.

This study has two main purposes. The first is to scrutinize the method of using correlation with the HEC to evaluate surrogate measures. We suggest additional aspects to consider in an evaluation. The second purpose is to show that some of the surrogate measures are mathematically equivalent. We show some consequences of this equivalence and question the comparison between the measures, and most importantly the use of several measures within the same study. Here we would like to concur with Rössner et al. [15] in their aim to “limit further comparisons between these fasting indices”.

We define the two most common measures of correlation, and discuss their characteristics and relation. In the next section we discuss why correlation measures alone are not suitable for method comparison. Further, we suggest other aspects worth considering. Next, we focus on three common surrogate measures; HOMA-IR, QUICKI and FIRI. We argue that they are one-to-one transformations of each other and discuss the consequences of this relationship. Finally some conclusions end the paper.

## Measures of correlation

A correlation coefficient measures the extent to which two variables tend to change together, both regarding strength and direction of the relationship. A number of different measures of correlation exist, the two most common being the *Pearson’s correlation coefficient* and the *Spearman’s rank correlation coefficient*.

The Pearson’s correlation coefficient measures the strength and direction of the *linear* relationship between two variables. It ranges from − 1 (perfectly linear negative relationship) to 1 (perfectly linear positive relationship). The Pearson’s correlation coefficient for a sample of data is defined as

1 $$ r=\frac{\sum_{i}(x_{i}-\bar{x})(y_{i}-\bar{y})}{\sqrt{\sum_{i}(x_{i}-\bar{x})^{2}}\sqrt{\sum_{i}(y_{i}-\bar{y})^{2}}},  $$

where (*x*_*i*_,*y*_*i*_) are observations of variables (*X*_*i*_,*Y*_*i*_).

**Fig. 1:**
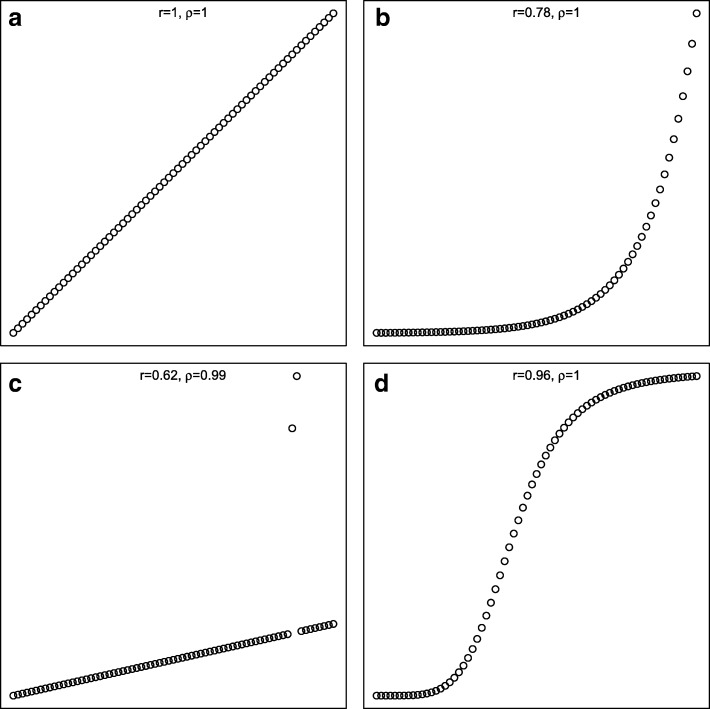
Simulations with corresponding correlations. **a** Linear. **b** Monotonic. **c** Outlier. **d** Monotonic

Standard tests and confidence intervals for the Pearson’s correlation coefficient rely on the assumption that data come from a bivariate normal distribution, possibly with unequal variances for *X* and *Y*, but with equal variances for all range of *X*- and *Y*-values. It is a measure which is sensitive to outliers, hence one or two “odd” measurements might influence the coefficient severely (Fig. 1c). Furthermore, Pearson’s correlation coefficient is by definition a measure of linearity, Hence, the coefficient is 1 in Fig. 1a, while it is only 0.78 in Fig. 1b in spite of a perfect monotone relation.

The dependence on linearity and outliers is reduced by ranking the variables before applying Formula (1), which leads to the Spearman’s rank correlation coefficient.

The Spearman’s rank correlation coefficient is a non-parametric measure of association between two variables. It measures the strength of a *monotonic* relationship between paired data. It ranges from − 1 (perfect monotonic decreasing relationship) to 1 (perfect monotonic increasing relationship). Note that a linear relationship is always monotonic (Fig. 1a), whereas a monotonic relationship is not necessarily linear (Figs. 1b, d).

A majority of studies base their evaluation of surrogate measures on strength of linearity with the HEC, i.e. on Pearson correlation. Less emphasis is put on strength of a joint trend not necessarily linear, i.e. on Spearman correlation. The choice of correlation coefficient should be based on the type of association that is of relevance. Does a trend necessarily have to be linear?

Correlational meta-analyses combining different coefficients sometimes follow an approach proposed by Rupinski and Dunlap [17] for converting Spearman correlations (*ρ*) to Pearson correlations (*r*):

$$r=2\sin\left(\rho\cdot\frac{\pi}{6}\right). $$

One should be aware that this formula relies on the assumption of a bivariate normal distribution. Most insulin sensitivity measures have skewed distributions, why this assumption is likely to be violated. Converting a Spearman correlation coefficient to a Pearson correlation coefficient for data which does not fulfill this assumption may give unreliable results.

## Evaluation of surrogate measures of insulin sensitivity

Investigators often wish to estimate insulin sensitivity with a simple, cheap and low-invasive method. Unfortunately, the true value of insulin sensitivity is not available to calibrate on. Thus, the usual practice is to evaluate the surrogate measure by comparison with an established technique, for instance the HEC. Correlation is the most commonly used method for this comparison. Correlation tells us something about the surrogate measure’s relationship (linear or monotonic) with the reference method. However, the information we gain from correlation is limited.

There are a number of factors influencing the correlation coefficient that one should be aware of. The *range* of measurements has a considerable effect on the size of the correlation coefficient. A larger range of measurements will generally give a higher correlation. Expressed differently, correlation will increase if the between subject variability increases, which may seem counter-intuitive. Furthermore, in particular regarding Pearson correlation, *outliers* have a high impact on the coefficient, as was seen in Fig. 1c. In addition, standard methods used for testing and estimation are influenced by *heterogeneous variance*.

We advice against relying too heavily on correlation. Instead we would like to encourage the use of additional methods of evaluation. In the remainder of this section we focus on additional aspects worth considering in an evaluation of a surrogate measure of insulin sensitivity.

In a more complete evaluation of any clinical measure, different aspects of variability should be considered; for instance repeatability, reproducibility and bias. *Repeatability* is a measure of how well a measurement can be repeated under identical conditions and is derived from the within subject variability of replicates. *Reproducibility* is a measure of how well a measurement can be replicated under differing conditions, e.g. with different observers, times and laboratories. A highly reproducible measure enables comparison between studies.

Surrogate measures of insulin sensitivity are in general not expected to give the same values as the measurements received from the HEC method. Due to the different natures of the insulin sensitivity measures, they tend to be on quite different scales and have different units. Thus, we do not necessarily strive for agreement between the surrogate measure and the HEC measurements, i.e. that the two measurements made on the same subject are close. Therefore, a bias (i.e. a systematic difference between the measurement methods) that is constant over the whole range of measurements is not an issue. However, a *bias that varies* over the range of measurements could be a problem. If, for instance, small values are underestimated while large values are overestimated for the surrogate measure, statistical testing might show significant differences between groups for the surrogate measure but not for the HEC.

Depending on the purpose of the study, it might be important to consider how well a measure can *discriminate* between groups of subjects, for instance healthy and diabetics, and to give the same result as the HEC in a statistical test situation. One way of evaluating the discriminatory ability is through ROC curves, which will be discussed in the next section.

As is becoming apparent, the definition of a perfect surrogate measure is not straightforward, a question addressed by Berger [18] among others. What makes a perfect surrogate depends on the purpose of the study. In the terminology of Buyse and Molenberghs [19], a perfect surrogate at the *trial level* is one which enables prediction of the treatment effect on the reference method from the treatment effect on the surrogate. A surrogate is perfect at the *individual level* when there is perfect association between the surrogate and the reference method, after correction for the treatment effect.

Several quantities have been proposed in the validation of a surrogate with respect to a reference method for a specific treatment. Freedman et al. [20] propose quantifying the *proportion explained* (PE), the proportion of the effect of treatment on the reference method that can be explained by the treatment effect on the surrogate. Buyse and Molenberghs [19] propose quantifying the *relative effect* (RE), the effect of treatment on the reference method relative to the effect of treatment on the surrogate. It should be noted that a large number of observations generally are needed for these validation procedures.

In summary, we find that correlation with the gold standard is not enough to evaluate a surrogate measure of insulin sensitivity. Although a valuable contribution to an evaluation, other aspects should be investigated, for instance repeatability, reproducibility and discriminatory ability.

## Equivalent insulin sensitivity measures

As mentioned previously, a large number of studies can be found in the literature comparing different surrogate measures. The evaluation is predominantly based on correlation with the HEC or some other reference method. The fasting surrogate measures HOMA-IR and QUICKI are frequently subject to this type of comparison. Some studies find that QUICKI has a higher correlation with the HEC, while others conclude that HOMA-IR is better. However, HOMA-IR, QUICKI (and FIRI) are equivalent in a mathematical sense. If two measures have a one-to-one correspondence, that is if each value for one has a unique counterpart in the other, then one of them is redundant in the sense that it can be transformed into the other. This is the case for HOMA-IR, FIRI and QUICKI. Pairwise, they are each strictly monotone functions of each other for any values of fasting insulin and glucose (see formulas in Table 2). As a result of the one-to-one correspondence a number of analysis methods will give equal results, which we demonstrate below. This should be kept in mind when evaluating these surrogate measures.

**Table 2:** Transformations between HOMA-IR, QUICKI and FIRI

Measures		
HOMA-IR and	HOMA-IR=FIRI ·(25/22.5)	FIRI=HOMA-IR
FIRI		·(22.5/25)
HOMA-IR and	HOMA-IR =*e*^1/*QUICKI*^/22.5	QUICKI =1/(log*HOMA*−
QUICKI		*IR*+log22.5)
FIRI and QUICKI	FIRI =*e*^1/*QUICKI*^/25	QUICKI =1/(log*FIRI*
		+log25)

### Equal Spearman correlations with the HEC

HOMA-IR and QUICKI have a Spearman correlation of − 1. Thus, their Spearman correlations with any other variable, e.g. HEC measurements, will apart from a minus sign be equal. However, HOMA-IR and QUICKI do not have a linear correspondence (unlike HOMA-IR and FIRI), why their Pearson correlations to HEC measurements will differ.

**Fig. 2:**
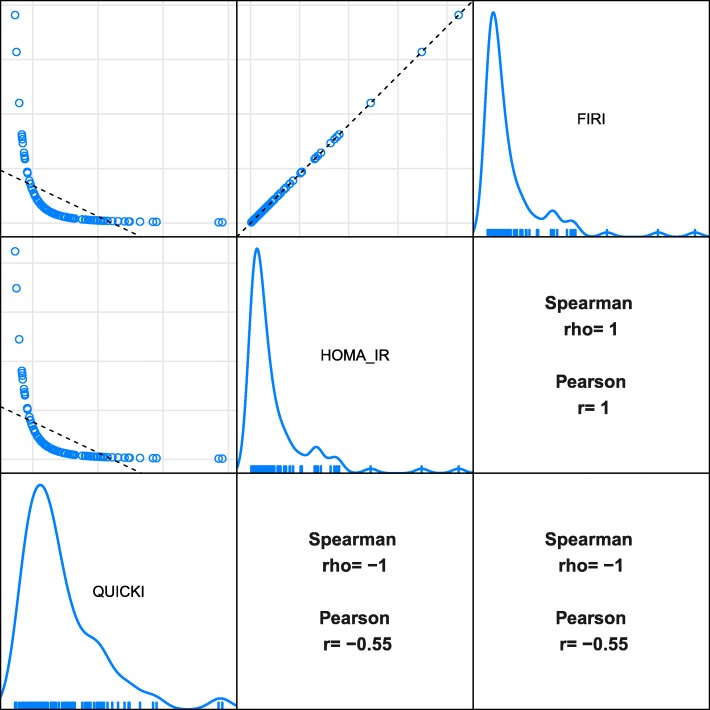
Simulations of QUICKI, HOMA-IR and FIRI

To exemplify, we simulated values of fasting glucose (assumed lognormally distributed, parameters from Cheng et al. [21]) and fasting insulin (assumed lognormally distributed, parameters from Li et al. [22]) in order to calculate QUICKI, HOMA-IR and FIRI (available in Additional file 1). In Fig. 2 we see scatter plots of the simulated surrogate measures with corresponding Spearman and Pearson correlations between them. You can see that there are perfect monotonic relationships between all three measures. Thus, the Spearman correlations are 1 or − 1 because of the monotone relationships, while the Pearson correlations are lower due to nonlinearity.

**Fig. 3:**
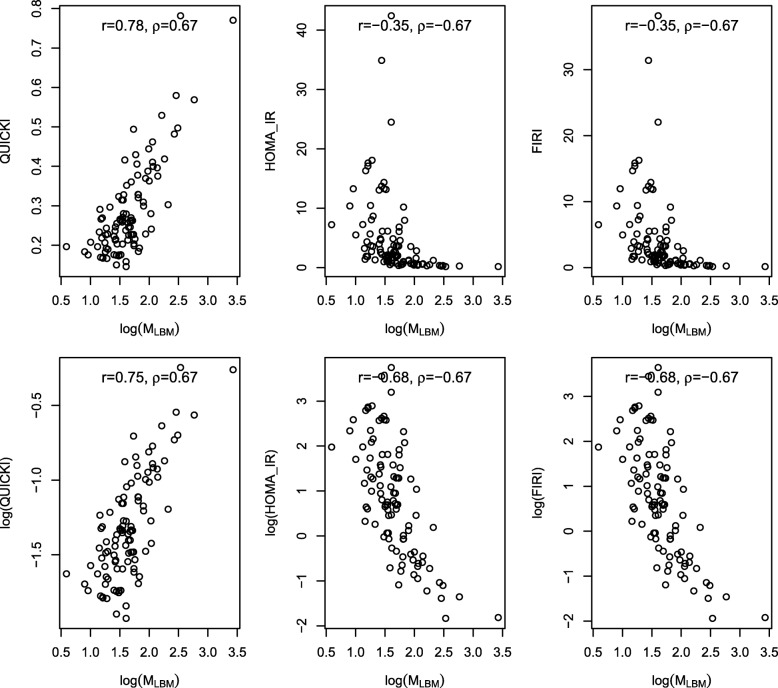
Scatter plots of QUICKI, HOMA-IR and FIRI against *M*_*LBM*_

*M*_*LBM*_ (assumed lognormally distributed, parameters from Duc Son et al. [23]) was simulated to correlate with QUICKI with a Pearson correlation of 0.75 (available in Additional file 1). Figure 3 displays scatter plots between the surrogate measures and *M*_*LBM*_. The Spearman and Pearson correlations between surrogate measure and *M*_*LBM*_ are also shown. The top three panels of Fig. 3 show QUICKI, HOMA-IR and FIRI plotted against the logarithm of *M*_*LBM*_. The bottom three panels show the logarithms of QUICKI, HOMA-IR and FIRI plotted against the logarithm of *M*_*LBM*_. Note that Spearman correlations are not affected by logarithmic transformations, whereas Pearson correlations are. Note also that as discussed above, QUICKI, HOMA-IR and FIRI have equal Spearman correlations with *M*_*LBM*_.

### Identical ROC curves

**Fig. 4:**
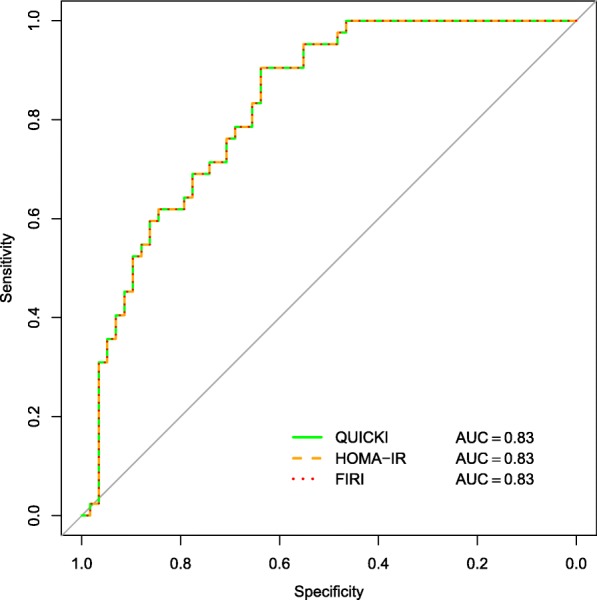
ROC curves of QUICKI, HOMA-IR and FIRI

Receiver operating characteristic (ROC) analysis is commonly used for evaluation of diagnostic ability. It is a tool designed to evaluate the performance of a classifier against a “true” binary classifier. In this setting the binary classifier could be healthy/insulin resistant (i.e. with decreased insulin sensitivity) as defined by the HEC. Figure 4 displays a ROC curve of the simulated dataset. We use the proposed definition from Bergman et al. [24] for insulin resistance as *M*_*LBM*_<4.7 mg/(kg min). The aim is to evaluate the discriminatory ability of the surrogate, i.e. how well the surrogate can distinguish between healthy and insulin resistant. For each value of the surrogate, the true positive rate is the percentage of insulin resistant who are diagnosed as insulin resistant by the surrogate. The false positive rate is the percentage of healthy who are diagnosed at insulin resistant by the surrogate. As pointed out by Rössner et al. [15], HOMA-IR and the inverse of QUICKI have equal ROC curves against a reference method. This is again due to the one-to-one correspondence. Any two variables with a monotonically increasing relationship will have equal ROC curves, regardless of classifier they are compared to. HOMA-IR, QUICKI and FIRI will thus always give identical ROC curves. We see that the curves for all the three methods are identical in the graph.

A statistical measure commonly retrieved from the ROC curve is AUC, area under the ROC curve. AUC has several interpretations, one of which is the probability that a random subject in one of the classes (here, a healthy subject) has a higher value of the surrogate measure than a random subject in the other class (here, an insulin resistant subject). For all surrogate measures in the simulated dataset AUC =0.83. Clearly, identical ROC curves give equal AUC’s.

### Identical results for nonparametric tests

As the monotonic relationship generates equal ranks, any order statistics, i.e. statistics based on ranks, will give identical results for HOMA-IR, QUICKI and FIRI. This includes many nonparametric tests but not parametric tests. Although based on the exact same information, i.e. the same values of insulin and glucose, parametric methods may give differing results for HOMA-IR and QUICKI.

Many studies have been reported where both HOMA-IR and QUICKI are used for parallel analyses. The results and conclusions are sometimes identical, sometimes different, depending on the statistical method used. If a rank-based method, such as a Mann-Whitney test, is used to compare the insulin sensitivity between two groups, by means of both HOMA-IR and QUICKI, the results will be identical, as discussed above. If, on the other hand, a parametric method such as a *t*-test, which rely on distributional assumptions, is used the results will differ to some degree, even with HOMA-IR log-transformed before analysis. This could be analysis of a treatment effect by comparing a treatment group to a placebo group. Often the results are similar, but we may fall within statistical significance for one measure and not for the other. An advantage of rank-based methods is that we avoid these types of problems. Bear in mind that a statistically significant result should be interpreted together with its effect size. To judge the clinical significance of a statistically significant finding, the estimated effect size should be compared to a threshold that is judged to be of practical importance.

Another note on the use of several indices for parallel analysis is that the information gained from using more than one measure from the same family of indices should be weighed against the risk involved with multiple testing.

In conclusion, we advise against further comparison between HOMA-IR, QUICKI and FIRI. We recommend choosing one of the three for analysis.

The same reasoning that has been applied to HOMA-IR, QUICKI and FIRI can be applied to the fasting surrogate measures *I*_0_/*G*_0_ and *G*_0_/*I*_0_. They also have a one-to-one and monotonic correspondence. They are thus equivalent in the same way that HOMA-IR, QUICKI and FIRI are.

## Conclusions

Correlation with the HEC is to date the most common method for evaluation of surrogate measures of insulin sensitivity. Correlation can give us information about the strength of the relationship with a reference method. However, as a method comparison tool correlation is inadequate, one reason being that the range of the measurements is crucial for the magnitude of the correlation coefficient. The measurement error, repeatability, reproducibility, and discriminatory ability are important aspects to be investigated and taken into consideration. Furthermore, the choice of correlation coefficient should be based on what type of relationship is of interest.

We have shown that HOMA-IR, QUICKI and FIRI are one-to-one transformations of each other. In many respects, e.g. Spearman correlations, ROC analysis and rank-based tests they are equivalent measures. We question the benefits of further comparison between these three measures. Our recommendation is to choose one of the three for analysis.

## Additional file

Additional file 1 Simulated values of HOMA-IR, QUICKI, FIRI and *M*_*LBM*_. (XLSX 10 kb)

## Abbreviations

AUC: Area under the curve
BMI: Body mass index
FIRI: Fasting insulin resistance index
*G*_0_: Fasting glucose
HEC: Hyperinsulinaemic euglycemic clamp
HOMA-IR: Homeostasis model of insulin resistance
*I*_0_: Fasting insulin
*M*_*LBM*_: Mean glucose infusion rate per kilogram lean body mass
NEFA; Non-esterified fatty acids; OGTT: Oral glucose tolerance test
PE: Proportion explained
QUICKI: Quantitative insulin sensitivity check index
RE: Relative effect
ROC: Receiver operating characteristic
